# Visualization of the biochemical dynamics of tannins, proteins, starch, and pectin in the endosperm and embryo of maize (*Zea mays* L.) during germination under salt stress and non-stress conditions

**DOI:** 10.1007/s00709-026-02173-2

**Published:** 2026-02-18

**Authors:** Öner Canavar, Huaicheng Li

**Affiliations:** 1 Faculty of Agriculture, Crop Science Department, Aydın Adnan Menderes University, 09100 Aydin, Türkiye; 2 Institute of Biology/Dahlem Center of Plant Sciences, Freie Universität Berlin, Berlin, 14195 Germany

**Keywords:** Corn (*Zea mays* L.), Seed germination, Metabolites changes, Embrio, Endosperm

## Abstract

Corn is one of the most economically important cereal crops cultivated around the world, and understanding the proper mobilization and utilization of starches, sugars, pectin, and metabolites in seeds is crucial for enhancing effective germination and promoting the development of healthy seedlings. Salt stress hinders seed germination by limiting water uptake, damaging cell structures, disrupting key metabolic processes, and impairing the function of endosperm and embryo tissues. However, visual representations and imaging of biochemical changes in the tissues and cells of the embryo and endosperm within corn seeds during germination under salt stress have been limited in the literature. In this study, we aimed to compare the structural changes and visualize the biochemical alterations occurring within the seed tissues (i.e., the outermost layer of the corn seed, endosperm, and embryo) at 6, 24, 48, and 96 h during germination under non-stress control conditions (0 dS m⁻¹) and salt stress conditions (4 dS m⁻¹ and 8 dS m⁻¹) using light fluorescence and scanning electron microscopy (SEM). We observed that pores between the pericarp cells (epidermis), the outermost part of the seed, did not shrink or deform under salt stress. In contrast, under non-salt stress conditions, the intercellular spaces between the pericarp cells opened rapidly. This limited porosity in the outermost layer cells could lead to inadequate water absorption, significantly contributing to delayed or failed germination under salt stress. Starch, the primary storage carbohydrate found in the endosperm, decreased rapidly under non-salt stress conditions; however, starch degradation slowed under salt stress. Almost no starch grains were detected in the embryo or scutellum cells. Protein depletion in aleurone cells occurred rapidly during germination in the absence of salt stress but slowed as salt stress increased. We also observed that the movement of metabolites from the embryo to the root and shoot regions within the seed was slowed or diminished under saline conditions. Alterations in cellulose and tannins were observed during seed germination under both salt and non-salt stress conditions; however, the scutellum cell walls retained their structural integrity due to partial, localized degradation. During germination, endosperm cell walls became more distinct, with an increased presence of pectin and mucopolysaccharides. The shoot region, initially rich in proteins, sugars, tannins, and pectins, was also found in the mesocotyl cells and intercellular spaces. Visualizing these changes in seed structure throughout our study enhances our understanding of the adverse conditions.

## Introduction

Salt stress has several negative effects on seed germination and growth of plant (Tarchoun et al. 2022). The two critical stages of plant growth are seed germination and seedling establishment. These stages are particularly sensitive to environmental conditions, including salinity. Seeds are typically sown in the upper soil layer, which may contain elevated salt concentrations, particularly in dry regions characterized by high evaporation, limited leaching, and intensive irrigation. These conditions lead to salt accumulation at the soil surface (Cuevas et al. 2019; Das et al. 2020). Salt stress creates an environment that hinders seeds from absorbing water from the soil, which is essential for initiating the germination process. Hence, without adequate water, seeds may either fail to germinate or germinate at a significantly slower rate (Ismail and Horie 2017; Lu and Fricke 2023). High salt levels in the soil can cause osmotic stress, a condition in which the external environment is more saline than the internal environment of the seed. This imbalance impedes the movement of water into the seed, leading to dehydration and inhibiting germination (Blum 2017; Fu and Yang 2023). Excessive salts, particularly sodium (Na⁺) and chloride (Cl⁻) ions, can also accumulate in various parts of seed tissues, resulting in ionic toxicity (Alharbi et al. 2022). This accumulation of ions can damage cellular structures and disrupt normal cellular functions, further hindering the germination process (Munns et al. 2006). Nikolić et al. (2023) explained that even if seeds can germinate under salt stress, the process is often delayed. This delay can result in uneven seedling emergence, which may negatively impact the overall growth and yield of crops. Salt stress can inhibit the enzymatic activities and metabolic processes that are essential for breaking down stored food reserves in the seed. This inhibition can deprive the seedling of the energy required for growth, thereby hindering or delaying germination (Guo et al. 2020; Sen and Puthur 2020).

Although the number of studies on the intricate physiological, morphological, and biochemical changes in various plants during seed germination has been gradually increasing, research on this complex process remains may be limited. The germination of seeds and the establishment of seedlings belongs to intense biochemical and very complex physiological and genetic activities (Zienkiewicz et al. 2014). In one of the studies, Wang et al. (2022) explained that maize BZ1 is a protein that is expressed specifically in the aleurone layer and is located in the nucleus. One imaging study using a microscope, Zhao et al. (2016), reported blue-stained dots of varying shades, representing starch granules accumulated in endosperm cells. Meanwhile, Ribeiro et al. (2020) observed red to purple hues indicating metachromasia, which is associated with a high pectin content in the structure. Zhou et al. (2023) microscopically analyzed the starches levels fluctuated notably from the stage of seed maturity to seedling establishment and consistently decreased during the germination stage of *Zostera marina* L. seeds stained with PAS (Periodic Acid-Schiff); however, the effects of salt stress on corn seed germination have yet to be fully elucidated. Further research is needed to understand the biochemical activities, changes, and regulation of energy metabolism in the various parts of the seed throughout the germination process of maize under salt stress.

There is a shift in carbohydrate metabolism, characterized by an increased mobilization of starch and stored lipids to provide energy during periods of stress. However, elevated salt levels may inhibit certain enzymes, such as amylase, which can slow down energy production (Liu et al. 2018). The endosperm serves as a source of energy and nutrients for the developing embryo; however, under salt stress, the breakdown of starch into sugars and proteins into amino acids may be inhibited or modified (Huang et al. 2021). Changes in lipid metabolism may also occur, impacting the levels of free fatty acids and other lipid-derived signaling molecules (Yi et al. 2025). Carbohydrates, amino acids, and organic acids exhibit altered profiles in response to stress, demonstrating a trend toward osmoprotective and energy-sparing compounds (McDonald 1999; Sun et al. 2007). There are some studies, which showed mobilization of carbohydrates, amino acids, and lipids from the endosperm to the embryo of rice (Galland et al. 2017), and enzyme secretion and nutrient breakdown in the aleurone layer (Xu et al. 2023), flow of metabolites (e.g., sugars, peptides) from the aleurone into the endosperm of barley (Aubert et al. 2018; Roustan et al. 2018). Lu et al. (2025) showed that SEM microscopic assessed morphology of endosperm and embryo cross-sections of maize microtissue morphology after chilling injury and HVEF (high-voltage electrostatic field) treatment during the germination of seed. Dean et al. (2021) investigated the migration patterns of oil bodies within embryo cells, various radicle tissues, and the epidermis and cortex cells of the radicle using light microscopy and transmission electron microscopy (TEM) during the acquisition of desiccation tolerance in chemically defoliated corn. Fu et al. (2023) studied the loss of function of ZmEREB92 and its effect on improved seed germination in maize plumula and radicle during imbibition using light microscopy. Ali et al. (2024) showed the visually the effects of salt stress on cytoplasmic organelles of white maize inbred line P4 in the root meristem cells using TEM micrographs. Although various microscopy methods have been employed in the literature to examine changes in the embryo, endosperm, and aleurone regions during the germination of maize and other plants, the imaging of metabolite changes in the cells during maize seed germination under moderate and intense salt stress conditions using different staining techniques has been limited. These stains are commonly used in microscopy to visualize and differentiate regions rich in specific metabolites within plant tissues. Their unique interaction profiles make them excellent tools for studying seedling development and metabolism. The Periodic Acid-Schiff (PAS) method is a widely used histochemical technique for staining carbohydrates and carbohydrate-rich structures, including glycogen, mucins, and glycoproteins (McManus 1948). This method is particularly valuable in plant histology for detecting polysaccharides, such as cellulose in plant cell walls, starch granules, and other carbohydrate-containing structures (Schmidt et al. 2009; Tüncü Konyar 2013; He et al. 2017). The Aniline Blue Black staining method is primarily employed to stain proteins, particularly in tissues where it is essential to differentiate between proteins and nucleic acid. It can reveal cell walls, proteins, and various cellular components in plant histology. Ruthenium red staining is a well-established histochemical technique utilized to stain acidic polysaccharides, including pectins and mucopolysaccharides, in plant materials (Waller et al. 2004). ‘It is particularly effective for visualizing plant cell walls because it binds to negatively charged components, such as pectic substances, which are abundant in the middle lamella of plant cells (Kohorn et al. 2021).^’^ Ruthenium red, a dye that binds to the carboxyl groups of acidic polysaccharides (such as pectins) and certain mucopolysaccharides found in cell walls, imparts a red stain to these structures. Ruthenium red detected pectins primarily in the outer periclinal walls, with intense staining around the entire exodermis cell and were also found in the primary wall of the endodermis and in the inner layers of its cell walls (Joca et al. 2020). Toluidine Blue stains the acidic components of tissue, including nucleic acids, pectins, and cell walls (Sridharan and Shankar 2012).

Under salt stress, maintaining a balance between metabolite production and utilization is crucial for seed germination, because plants are susceptible to the harmful effects of salinity at all stages of their life cycle, with the germination and seedling stages being the most vulnerable (Atta et al. 2023). Delayed or impaired mobilization of stored nutrients, increased demand for osmoprotectants, and the management of oxidative stress significantly influence the metabolic dynamics within these tissues. The issue of seed germination has also become increasingly problematic in saline soils worldwide. Corn’s also unparalleled versatility, high productivity, and global integration make it indispensable to modern agriculture, economies, and cultures. Therefore, the germination of corn, a costly seed for farmers, in saline soils requires thorough investigation.

The objectives of our study were: (1) to observe how salinity affects and alters the ultrastructural characteristics of seeds subjected to salt stress; (2) to visually investigate the migration of sugars, starches, and protein compounds in the embryonic cells of both seeds and roots during germination under salt stress; (3) to visually determine the differences in these changes caused by salt stress in maize seeds using light microscopy, fluorescence microscopy, and scanning electron microscopy. We also aimed to visually assess the changes in the structure of the cells in the outermost layer of maize seeds during a 96-hour germination period, both under salt stress and non-salt stress conditions, using scanning electron microscopy.

## Materials and methods

### Seed material and experimental design

Corn seed genotype “Kalumet” (KWS seed company©) was utilized as the plant material. Although the seed was certified seed, seed sizes were selected to be the same size before the beginning of germination and seeds of medium size were used for germination. Used a filter paper, folded it to fit inside container (Petri dish, 15 cm width). The seeds were placed on moist filter paper, both above and below them. Ensure that the seeds do not touch each other to prevent mold growth and to facilitate easy observation of each individual seed. Place the container with the seeds in a warm, dark location, as well as in a well-lit area, providing 12 h of light and 12 h of darkness in growth chamber. A temperature range of 22 degrees Celsius (°C) was generally recommended for seed germination; however, it was important to verify the specific requirements for the type of seeds in the air conditioning cabinet.

In the experiment, the control group was established with 0 dS m⁻¹ NaCl, while two different salinity environments were created at 4 and 8 dS m⁻¹ NaCl. Salt applications were administered using irrigation water. We calculated and adjusted the salt concentrations in the irrigation water for a total volume of 1,000 mL.

$$dS\;m^{-1}=10\;mM\;NaCl=0.584gL^{-1}$$

$$\begin{array}{ccc}For\;4\;dS\;m^{-1}&1\;L\;water\;4\;dS\;m^{-1}&2.336\;g\;NaCl\end{array}$$

$$\begin{array}{ccc}For\;8\;dS\;m^{-1}&1\;L\;water\;8\;dS\;m^{-1}&4.672\;g\;NaCl\end{array}$$

Fifty seeds were sown in each of the three environments. Daily irrigation was provided from the onset of germination, and the drying filter paper in the Petri dishes was regularly replaced to prevent salt accumulation and maintain consistent salt concentrations of 4 dS m^−1^ and 8 dS m^−1^. Petri dishes were replaced and watered daily with 10 mL of each salt concentration.

### Collection and preparation of samples

A sample was collected prior to germination to serve as a control before the germination process commenced. The second sampling was conducted 6 h after germination began, the third sampling occurred 24 h later, the fourth sampling took place 48 h later, and the fifth sampling was performed 96 h later, all under three different conditions (“0 dS m^− 1^ kontrol, 4 dS m^− 1^ and 8 dS m^− 1^ salinity). BNF Roti^®^-Histofix (Buffered Neutral Formalin) was used to maintain the viability of germinating seeds (ROTH, Germany), because “Roti^®^-Histofix” is a fixative solution, typically composed of formaldehyde, used in histology to preserve biological tissues for microscopy by stabilizing their structures. This approach ensured the consistency of the sample at the time of collection by effectively killing all cells and tissues. All samples were washed in a Falcon tube with 1% PBS for 60 minutes initially, followed by two brief washes.

###  Preparation of the samples for scanning electron microscopy

Germinating whole seeds were initially rehydrated with ethanol (EtOH) and then immersed in 2% osmium tetroxide (OsO4) (Carl Roth) for 90 min. They were then washed with water 10x time. All materials were dehydrated through a series of increasing ethanol dilutions, ranging from 10% to 100%, and then transferred to acetone for several hours (both from VWR) before undergoing critical point drying with a Leica EM CPD 300 (Leica, Germany). All corn seed samples were mounted on aluminum pin stubs (Agar Scientific Ltd, UK) using nail polish (Maybelline, USA) and subsequently gold-coated with an EM ACE 200 sputter coater (Leica). Evaluations and observations of the samples were conducted using an EVO MA10 scanning electron microscope (Zeiss, Germany) with a beam current set to 20 kV and 2.56 mA.

### Preparation of the samples for light and fluorescence microscopy

After the BNF treatment of the germinating maize seeds, they were dehydrated using a gradient of ethanol, ranging from 10% to 100%. The samples in glass tubes were stored at 4 °C by adding 100 mL of Technovit 7100 hardener. The infiltration solution for embedding was prepared by mixing Technovit 7100 and Technovit 2 in a 1:10 ratio. The samples were placed in a small plastic container with 3 mL of this solution for 40 min to allow for hardening. For light and fluorescence microscopy, the material was embedded in 2-hydroxyethyl methacrylate resin, Technovit 7100 (Kulzer GmbH, Germany), in accordance with the manufacturer’s instructions. Serial longitudinal sections of germinated seeds were made at different thicknesses (10, 12 μm) using a Leica Autocut rotary microtome (Leica) with disposable blades (Faust, Germany).

### Staining of samples

Sections of the lamella were also stained with 1% aqueous ruthenium red (Acros Organics, USA) to detect pectin, tannins, and other soluble carbohydrates, and/or with Periodic Acid-Schiff’s reagent (Carl Roth GmbH, Germany). Reaction to detect degrageted starch grains and other insoluble carbohydrates. 15% acetic acid solution containing 2,4-dinitrophenylhydrazine (2,4-DNP), 1% periodic acid, Schiff’s reagent, and 0.5% sodium metabisulfite in 1% hydrochloric acid (HCl) was used for staining with the periodic acid-Schiff (PAS) method. After being immersed in acetic acid for 20 min, the samples were rinsed in running tap water for 1 min and then soaked in water for an additional 20 min. They were then transferred to periodic acid for 10 min and subsequently washed with tap water for 20 min. The samples were subsequently treated with Schiff’s reagent for 10 min, followed by exposure to sodium metabisulfite in hydrochloric acid for 6 min. Finally, the samples were washed with water and dried.

Aniline blue black dye (typically 0.1% solution), fixative (e.g. Roti^®^-Histofix or formalin), 7% acetic acid (for cleaning), mounting medium, distilled water were used. A 1% Aniline Blue Black solution was prepared in acetic acid, typically using 7% acetic acid. All samples were immersed in a 1% aniline blue-black solution for 6 to 7 min at 65 °C in the oven. The stained sections were rinsed in 7% acetic acid and distilled water for a few minutes, if the water-based method was used, to remove any excess dye.

For all samples, the tissue sections were immersed in a ruthenium red solution for 5 min at room temperature. After staining, rinse the tissue sections with distilled water to remove any excess dye.

Stained samples were subsequently stained with 0.05% aqueous toluidine blue (Carl Roth). Depending on the type and thickness of the tissue, the sections were stained in a toluidine blue solution for 30 s. The stained sections were thoroughly rinsed with distilled water three times to remove the dye. Then, dehydrated the tissue by passing it through increasing concentrations of alcohol (70% for 2 times, and 100%). Calcofluor is a fluorescent dye utilized for staining various components in biological samples, particularly in plant tissues (Picinini et al. 2024). Orimine “Acridine orange” is a nucleic acid-selective fluorescent dye used to differentiate between DNA and RNA in plant and animal cells (Damas-Souza et al. 2019). A 0.1% (w/v) Calcofluor stock solution was already prepared by dissolving 0.01 g of Calcofluor powder in 10 mL of distilled water, followed by thorough mixing according to the manufacturer’s instructions and storage at 4 °C protected from light. A 0.01% (w/v) Acridine Orange working solution was already prepared by dissolving 0.001 g of Orimine powder in 10 mL of distilled water and was protected from light until use. Equal volumes of Calcofluor White (0.1%) and Orimine (Acridine Orange, 0.01%) —250 µL of each—were pipetted into a single tube and gently mixed to prepare a 1:1 staining solution. A measured volume of this mixed stain was then applied to the sample and incubated for 5 min in the dark. After incubation, the sample was thoroughly rinsed with distilled water to remove excess stain (Grossniklaus et al. 2003; Gao et al. 2020).

The sections stained with various solutions were examined using a ZEISS Axio Imager M2, coupled with an Axiocam 506 digital camera (ZEISS, Oberkochen, Germany) and equipped with differential interference contrast illumination.

## Results

### Development of the radicle and changes of the intercellular space of pericarp cell (SEM)

**Fig. 1:**
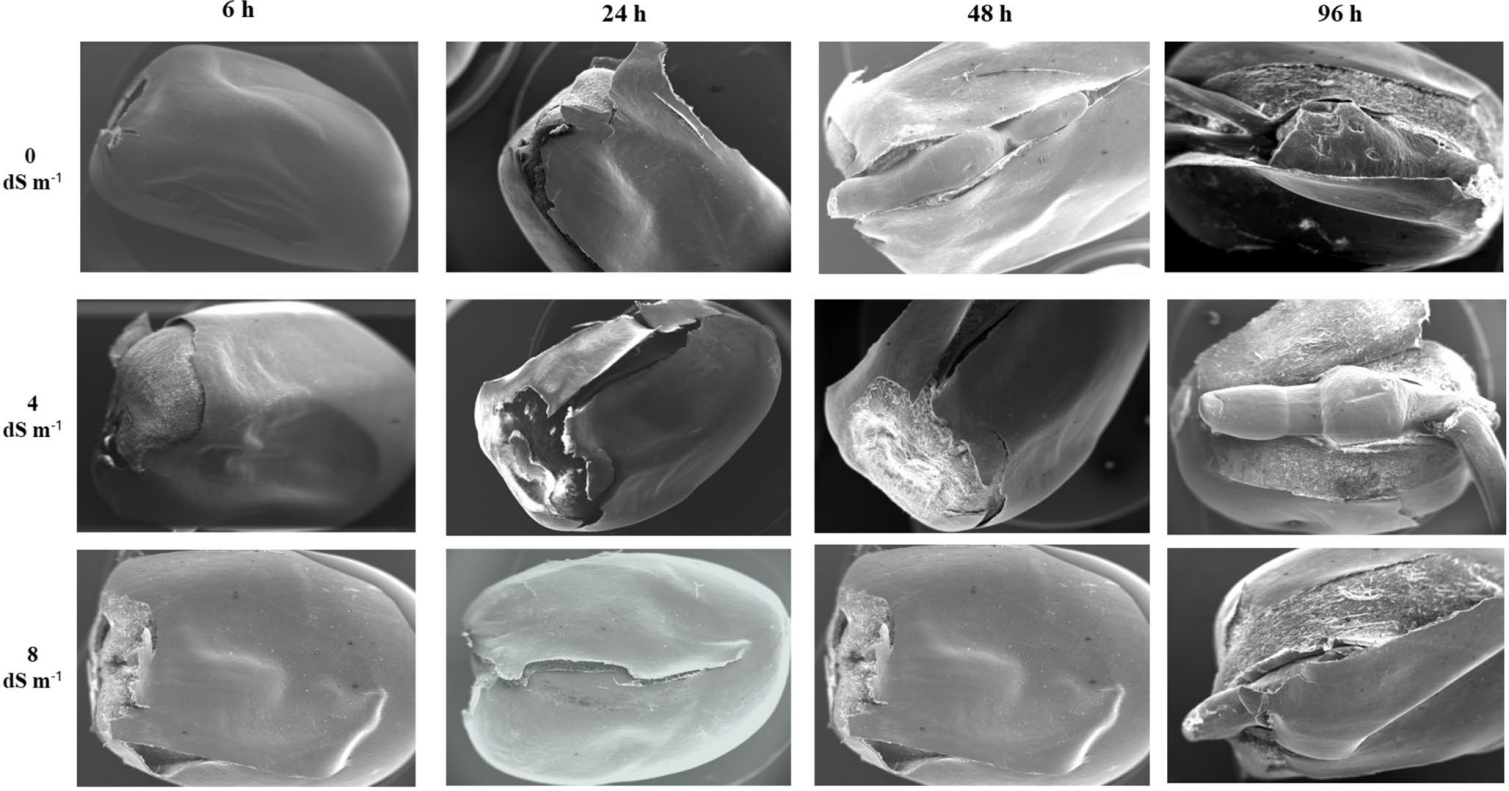
Scanning electron microscope (SEM) images of whole maize seeds under 0, 4, and 8 dS m⁻¹ salt stress conditions, captured at 28× magnification at 6, 24, 48, and 96 h after the beginning of germination

**Fig. 2:**
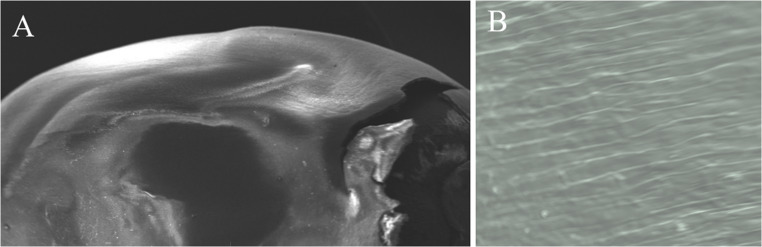
SEM microscope image of the testa seed coat of a maize seed before germination (A: 28X magnification and B: 330X magnification, respectively)

**Fig. 3:**
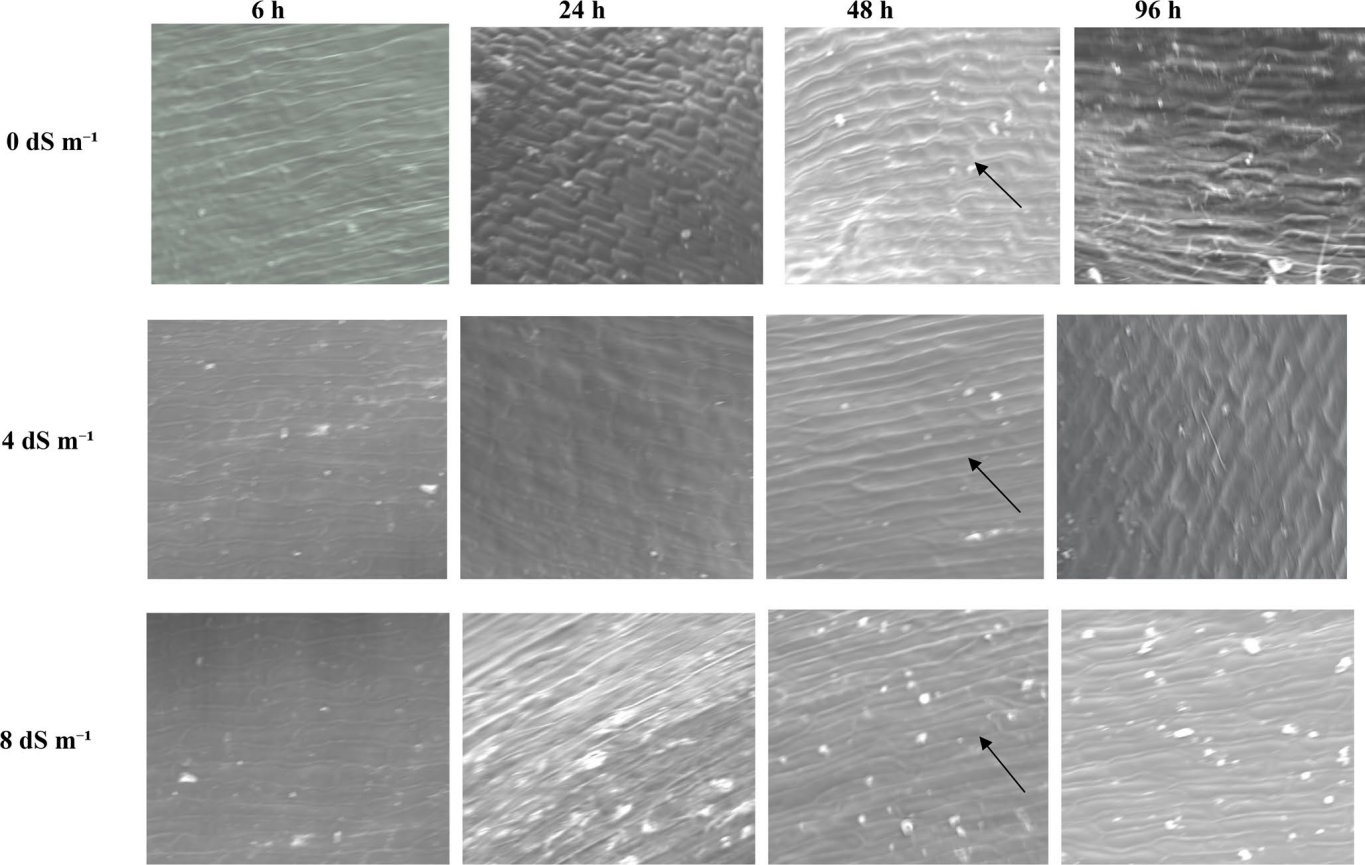
A scanning electron microscope (SEM) image of testa (pericarp) cells from the seed coat in a region near the micropylar area of maize under salt stress conditions of 0, 4, and 8 dS m⁻¹. The images were captured at 300–330X magnification at 6, 24, 48, and 96 h after the beginning of germination **6 h 24 h 48 h 96 h**

**Fig. 4:**
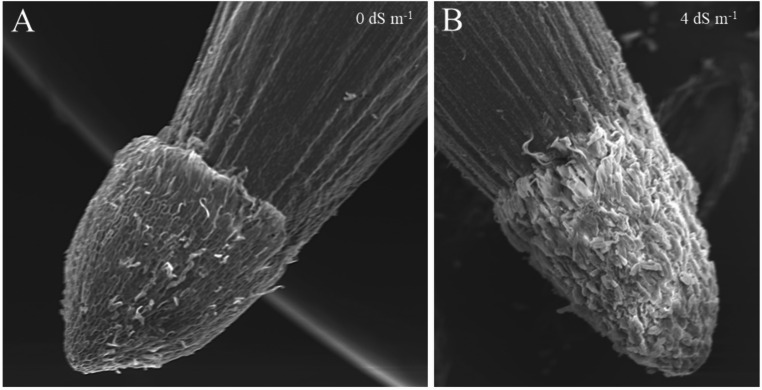
A scanning electron microscope (SEM) image at 150X magnification shows the root structure of seeds under A: 0 dS m^− 1^ non-stress conditions and at B: 4 dS m⁻¹ salt stress after 96 h of germination

Our study demonstrated that the scanning electron microscope (SEM) provided high-magnification observations of seed germination, offering detailed insights into the structural changes that occur during this process (Figs. 1, 2, 3 and 4). Scanning electron microscopy (SEM) revealed high-resolution images of the smooth pericarp, which serves a protective function before water uptake (Fig. 2). As germination begins, the intercellular space between the pericarp cells (epidermis), which was the outermost part of seed coat begon to crack, split, porosity, the breakdown of protective layers could be observed in scanning electron microscope (SEM) images (Fig. 3). The SEM images showed that radicle expansion, crucial for germination, occurs earlier under normal conditions, with seed coat disintegration starting after 24 h. Under salt stress, these changes are delayed (Fig. 1). Although random selections were generally made from the sown seeds, Fig. 1 illustrated that under extreme salt stress conditions, such as 8 dS m⁻¹, a small radicle was visible; however, in half of the seeds, this radicle did not fully emerge from the tip cap. High levels of salt (8 dS m-1 NaCl treatment) caused delayed or uneven absorption of water, leading to irregular or incomplete cracking of the seed coat (Fig. 1). Damaged or misshapen radicles may exhibit fewer root hairs (coleorhiza cells on root cap) or irregular cell structures shrunken or wrinkled, as observed in Fig. 4, because of indicating dehydration due to osmotic stress. In seeds that exhibit poor or delayed germination under salt stress, scanning electron microscopy (SEM) revealed structural differences, such as abnormalities in the radicle, cell shrinkaged, adhesion of cell walls, and insufficient cell expansion (Fig. 3). This limited porosity among the testa cells could leaded to inadequate water absorption, which significantly contributed to the delayed or failured of germination under saline conditions. Since these breaks tend to be more pronounced in the vicinity of areas such as the tip cap or in regions where the seed coat is thinner, we attempted to analyze the structure of the testa cells in a region closer to the micropylar area at approximately 300–350x magnification (Fig. 3).

### The differences of meristematic cells and root development compared between salt stress and Non-Salt stress

**Fig. 5:**
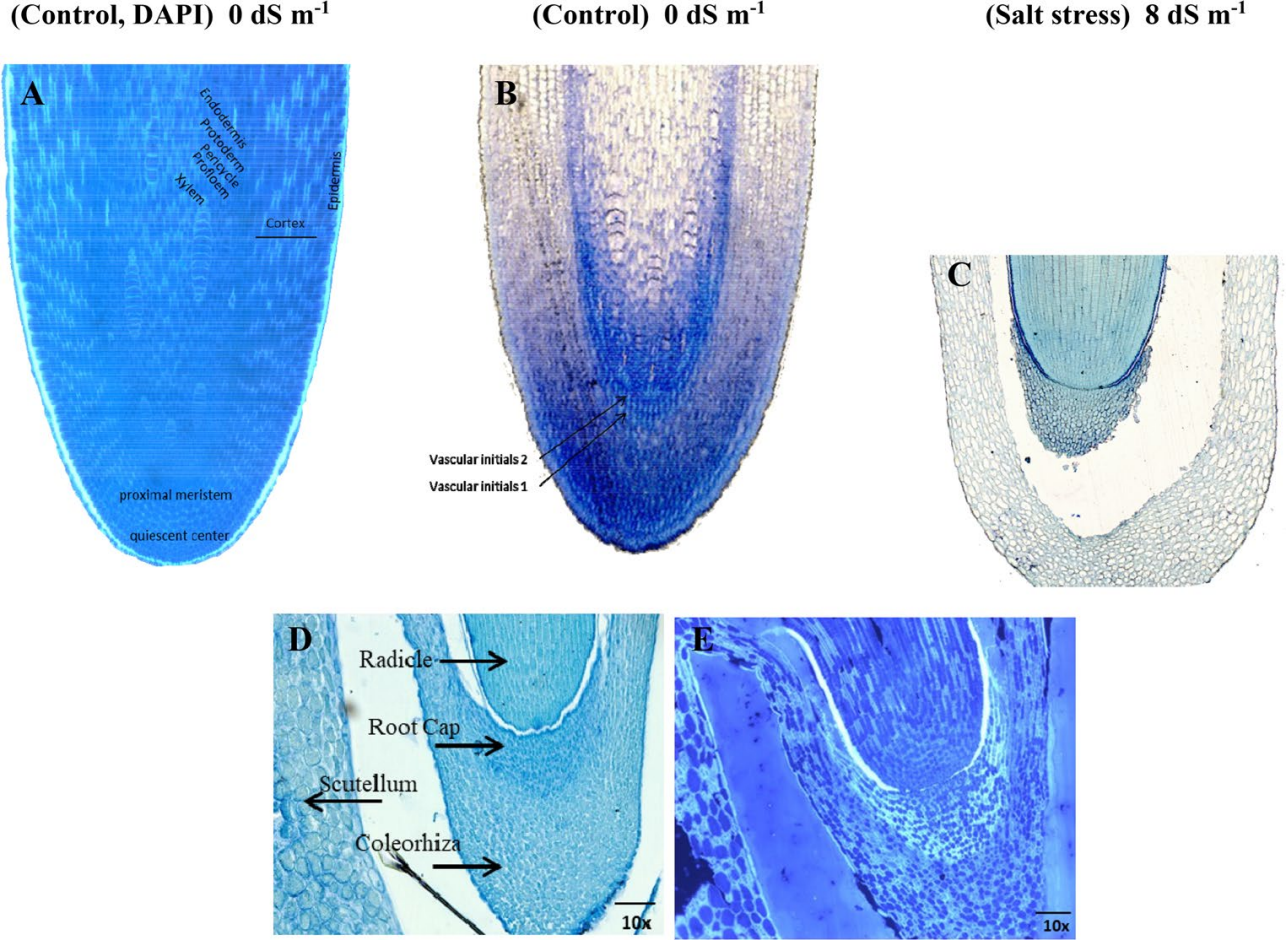
(A): The DAPI image and (B): The development of the root radicle and root structures of the embryo (stained with Toluidine Blue O) are shown just prior to the emergence of the radicle, at 96 h after the beginning of germination under non salt stress condition (0 dS m^− 1^). (C): still undeveloped radicle within the seed (stained TBO) under 8 dS m^− 1^ high salt stress condition. (D): stained TBO and (E): DAPI image; the image depicting salt stress was captured at 5x magnification. The two bottom images in the figure, which was captured at 10x magnification, illustrated the structure of the radicle at the stage before seed germination begins. All images, including those of the root and the seed, are cross-sectional samples taken precisely from the midpoint of root and seed. Scale bar: 100 μm

Both light microscope and fluorescence microscope images of sections taken from the center of the root illustrate the root cap of a seed germinated under salt stress (just prior to the emergence of its radicle) at 96 h (Fig. 5). It was evident in this study that the root cap of the germinated seed required time to separate from the coleorhiza under salt stress. In the samples prior to germination, it was observed that the root cap and coleorhiza were fused (Fig. 5, bottom of the figure). The most detailed image of the fusion between the root cap and columella was clearly visible in the DAPI-stained image (Fig. 5). In Fig. 5, it is particularly noticeable that the root caps of the roots emerging from seeds germinated for 96 h under non-stressed conditions have been removed. Under salinity stress, meristematic cells were especially susceptible, and their impairment significantly affected root development and overall seedling health (Fig. 5). In our study showed that the proximal meristem in roots was situated near the dormant center and comprises actively dividing cells (Fig. 5). Figure 5 showed that cell division in the proximal meristem was inhibited by elevated salt concentrations. Another significant finding in our study was that, although the seed radicle was not typically removed to outside under high salt stress conditions, such as 8 dS m⁻¹, the radicle within the seed developed a coleorhiza at the tip of the root cap for 96 h (Fig. 5).

### The biochemical structure of cells in the embryo (The middle of Schutullem) during germination

**Fig. 6:**
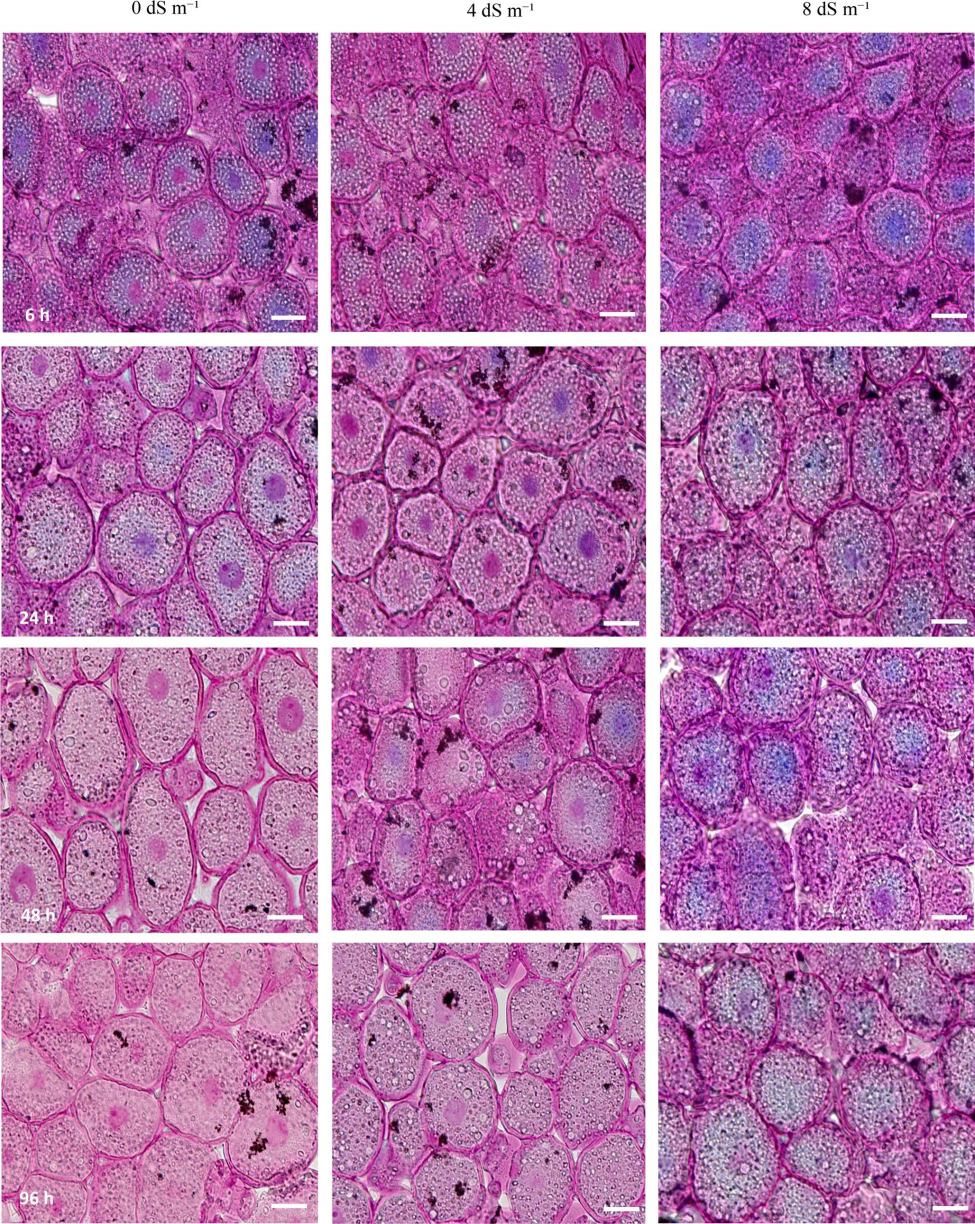
The structure of cells in the embryo (the middle of schutullem part in embryo) was stained with Ruthenium Red and Toluidine Blue O. The magnification: 40X. Bar: 10 μm

The cell walls of the schutullem part in the embryo loosensed and allowing for cell expansion to occured after 24 h at germination under non-stress condition (Fig. 6). Our study demonstrated that during seed germination, changes in cellulose and tannin levels within the cells were clearly observable under both non-saline and saline stress conditions (Fig. 6). It was clearly revealed in the microscopic image that the change in seed germination under salt stress conditions occurs very slowly (Fig. 6). Figure 6 showed that in contrast to pectin, cellulose is not fully degraded during germination. Figure 6 also showed also that cell walls could stretch without compromising their structural integrity, as the breakdown is partial and localized. Perhaps, this should be essential for allowing the radicle to penetrate the testa. While no differences were observed in the cell wall structures of the scutellum at the beginning of germination under varying salt stress concentrations, it was noted that in the later stages of germination, the cell walls in the scutellum of seeds not subjected to salt stress thinned and expanded more rapidly, accompanied by a further decrease in pectin content (Fig. 6). It could be concluded that, particularly under extreme salt stress conditions, the dissolution of cell walls in the scutellum occured very slowly, and there was minimal change in cell structures (Fig. 6). As illustrated in Fig. 6, it was determined that the tannins—believed to impart a bluish color to the scutellum cells at the onset of seed germination when stained with Ruthenium Red (RR) and Toluidine Blue O (TBO)—decreased progressively throughout the germination process. This reduction in tannin levels was observed to occur more rapidly under non-saline conditions. In contrast, under 8 dS m⁻¹ salt stress conditions, the tannins remained slightly more stable and did not show significant degradation quickly. This resulted in the formation of blue, purple, and pink colors in the nuclei of scutellum cells that were sequentially stained with both Ruthenium Red (RR) and Toluidine Blue O (TBO). Notably, in the absence of salt stress, the nuclei of the cells turned quickly pink after 24 h of germination (Fig. 6).

### The chances of starch and protein in embrio and endosperm cells

**Fig. 7:**
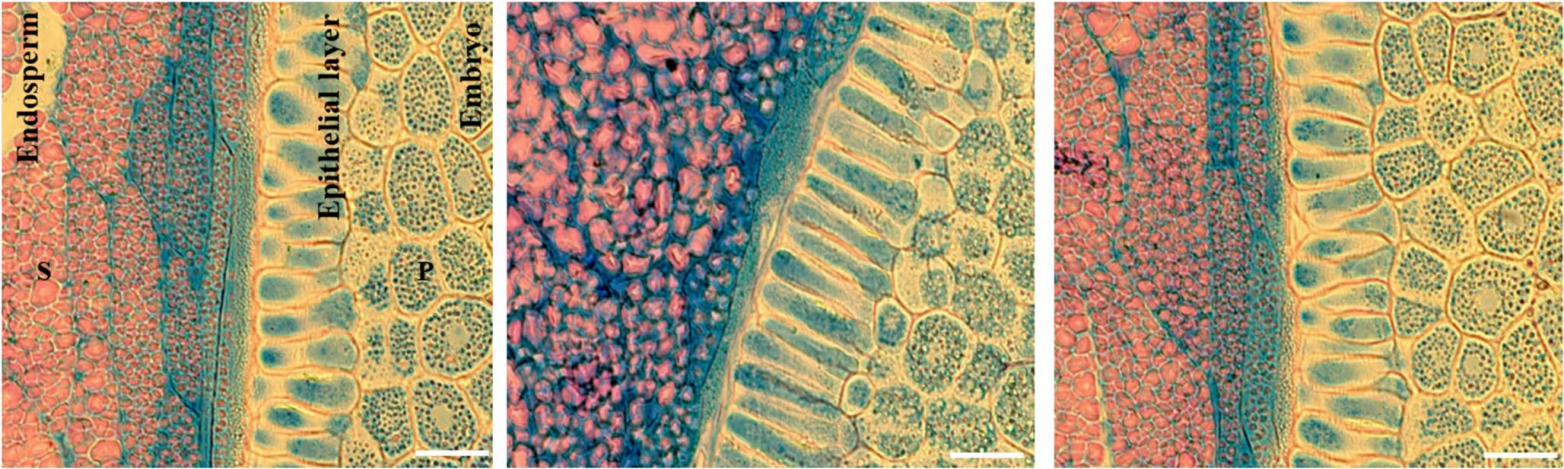
The cellular structures in the endosperm and embryo were stained with Aniline Blue Black and Periodic Acid-Schiff (PAS) reagents. The images were captured at 20× magnification. The sectioning samples were taken from seed under non-salt stress before germaination. (S): Strach granules (Pink color), (P): Protein drops (blue points in cells of the embryo part). Scal bar: 100 μm

Our study clearly demonstrated that starch stored in the seeds is localized in the endosperm, with no starch granules present in the embryo (Fig. 7). In the cross-sectional samples stained with both Aniline Blue Black and Periodic Acid-Schiff (PAS), specifically in the middle of the seed, the starch granules in the endosperm section appeared pink, whereas no pink coloration was observed in the scutellum (Fig. 7). The scutellum was predominantly blue, with protein spots detected in each cell (Fig. 7). Aniline blue-black staining also identified the cell nuclei within the scutellum. Additionally, proteins were identified in each epithelial layer aleurone cell located between the endosperm and the scutellum (Fig. 7). Small changes in the structure of the aleurone cell (Epithelilal layer) can also be observed, such as thinning or elongation (Fig. 7).

**Fig. 8:**
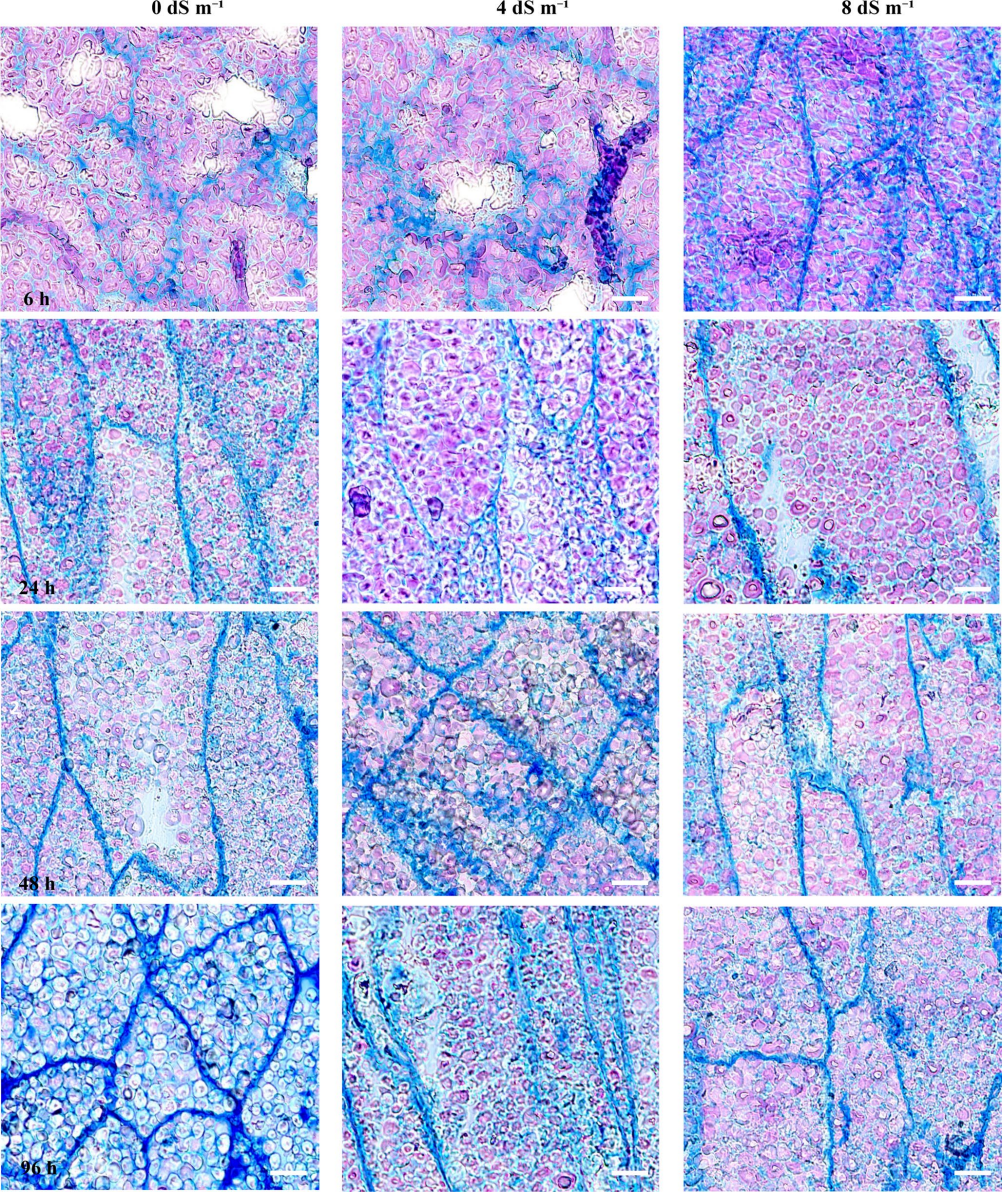
The cellular structure of the fixed endosperm was stained with Aniline Blue Black and Periodic Acid-Schiff (PAS). The samples are arranged from left to right, representing different salt stress conditions (0, 4 and 8 dS m^− 1^). Scale bar: 100 μm. Magnification: 10×

**Fig. 9:**
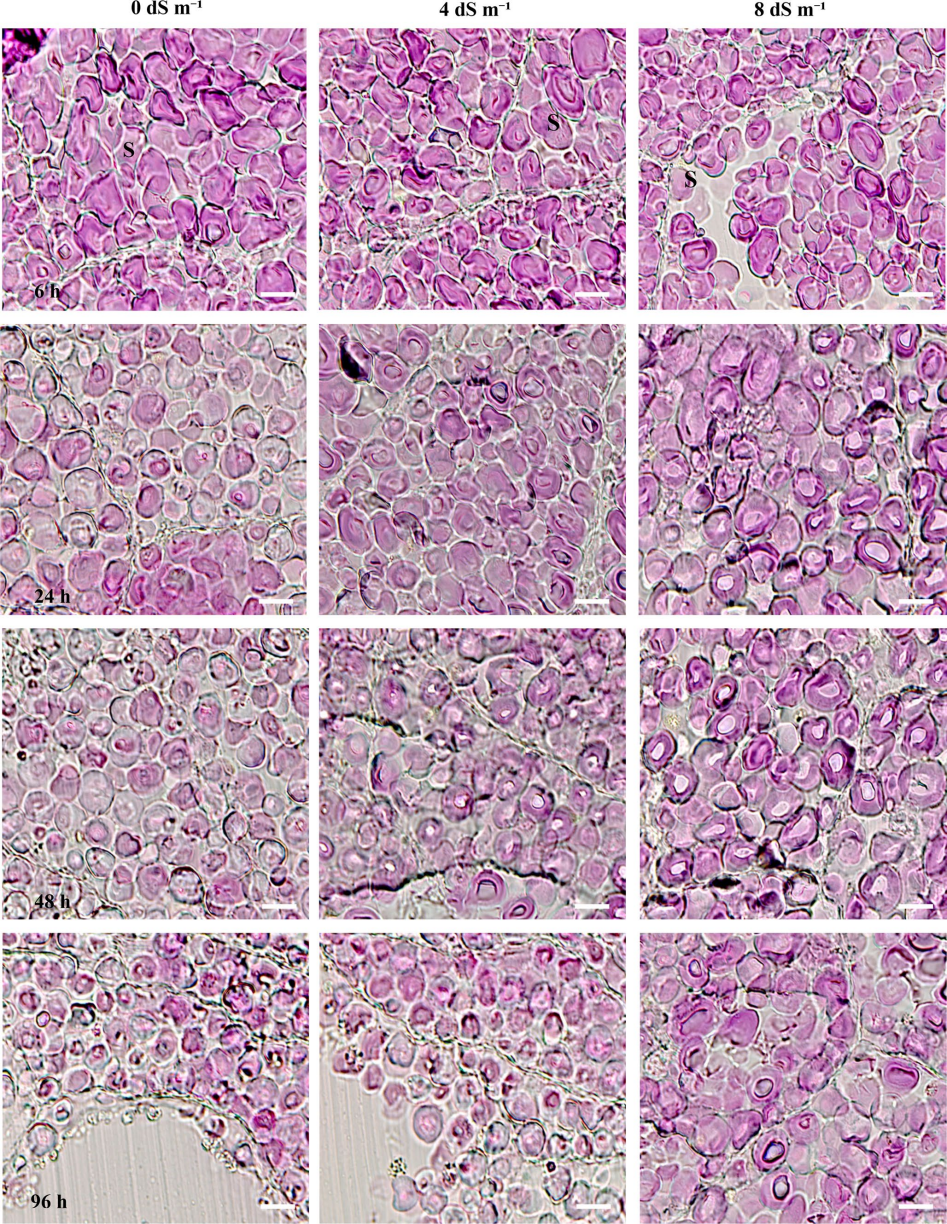
The cellular structure in the fixed endosperm tissue was stained exclusively with Periodic Acid-Schiff (PAS). The samples are arranged from left to right, representing different salt stress conditions (0, 4 and 8 dS m^− 1^). (S): starch granules (all pink-colored granules) in the endosperm. Scale bar: 100 μm. Magnification: 20×

In seed endosperms stained with both aniline blue-black and PAS, as well as with PAS alone, the starch content within the granules decreased, while the intensity of colors indicating sugar accumulation increased (Figs. 8 and 9). It was observed that starch degradation occurs at a significantly slower rate in seeds germinated under salt stress conditions (Figs. 8 and 9). Although we did not measure the size of the pink-colored granules, subtle structural changes were evident as germination progressed. These changes appeared less pronounced with increasing salt stress concentration. Notably, in endosperm cells stained only with PAS, the starch granules exhibited even greater shrinkage during germination (Fig. 9). In particular, Fig. 8 illustrated that during the germination stage, sections stained with aniline blue-black and PAS exhibit a pronounced and thicker blue coloration in the cell membranes and the intercellular spaces. However, this blue coloration on the cell membranes and between the cells was also observed under salt stress conditions, albeit at a slower rate. During germination at elevated salt concentrations, such as 8 dS m⁻¹, both the formation of starch granules and the development of blue coloration between cells occured at a significantly reduced pace (Fig. 8).

**Fig. 10:**
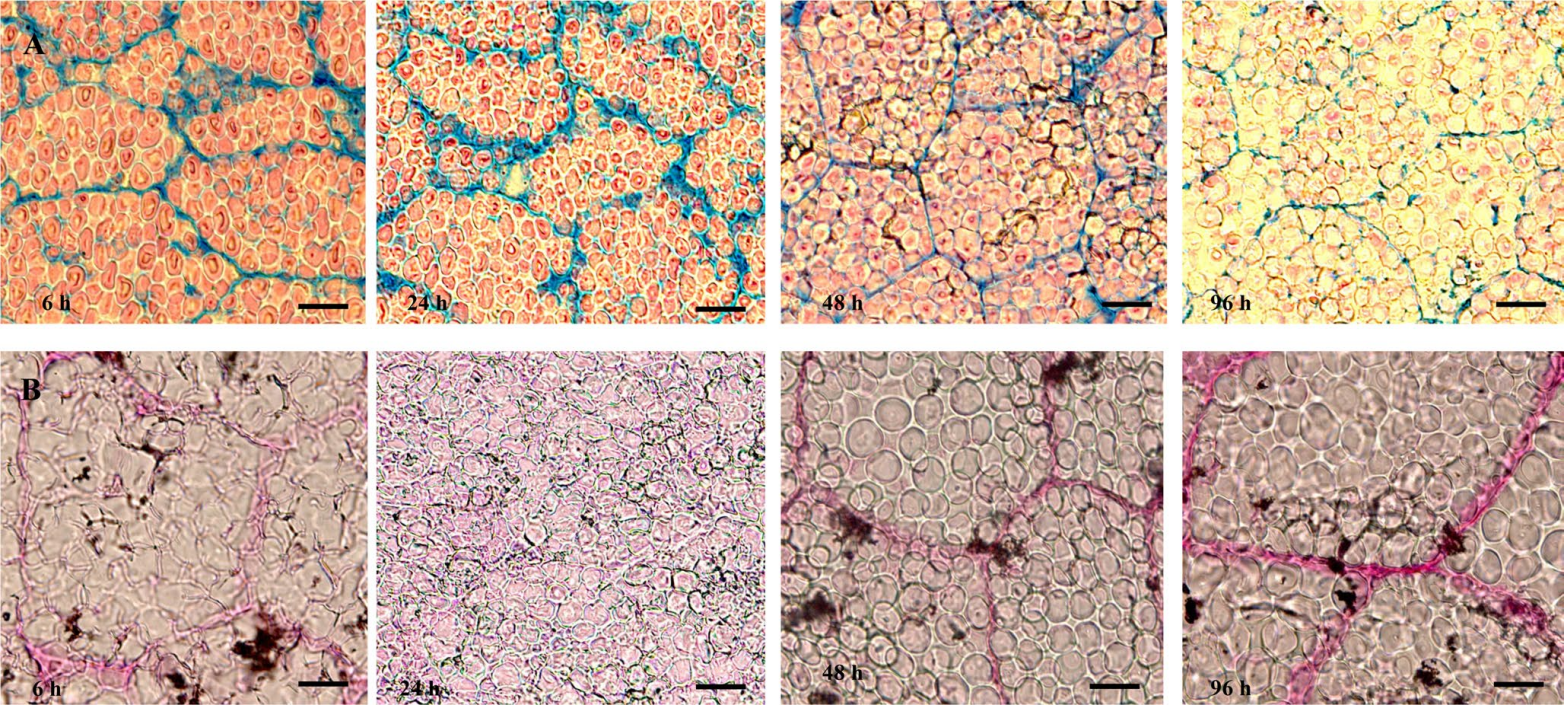
(A) Samples of the endosperm from the middle of seed cross-sections under non-salt stress condition (0 dS m^− 1^) were stained with PAS and Toluidine Blue O (20× magnification). (B) The bottom four samples were stained with Ruthenium Red and Toluidine Blue during the germination period (20× magnification). Scale bar: 100 μm

In our study, we performed staining with PAS and toluidine blue O, as well as with ruthenium red and toluidine blue O, on germinating seeds under non-salt stress conditions. This was done to confirm changes in starch granules within the cells and alterations in cell membranes (Fig. 10). In endosperm cells stained with ruthenium red and toluidine blue O, neither pink nor blue coloration was observed (Fig. 10B). No cell membrane structures were identifiable during the first six hours of germination (Fig. 10B). However, in the subsequent hours of germination, the structures of granules and cell membranes became clearly visible (Fig. 10). The most interesting result was that the cell walls of the endosperm cells in RR- and TBO-stained seeds became more pronounced as germination progressed, and pectin and mucopolysaccharides became more prominent (Fig. 10B).

### Variations between the coleoptile section of the mesocotyl and the onset of shoot development

**Fig. 11:**
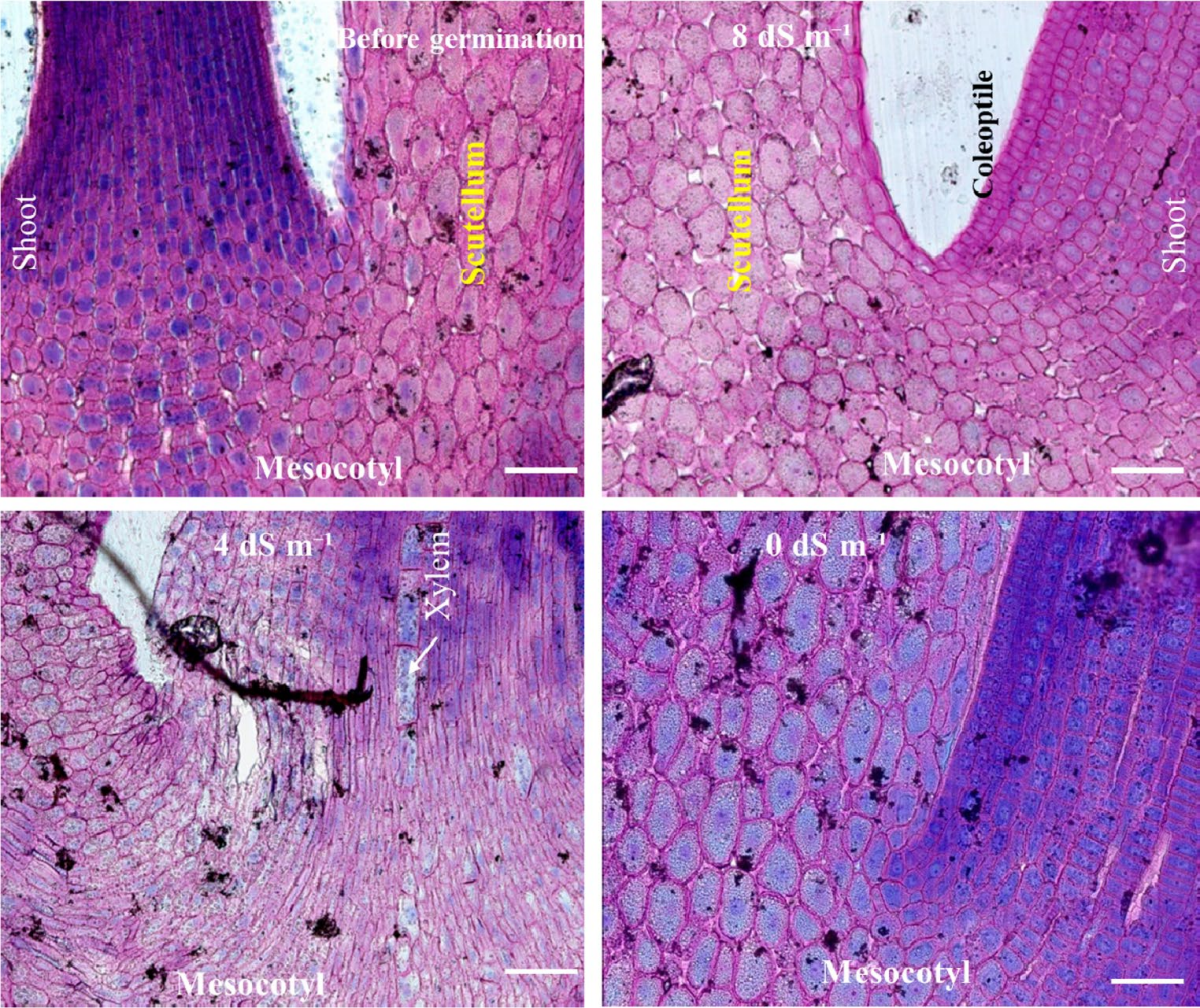
The cellular structure of the shoot and the embryo (scutellum), fixed in place, was stained with Aniline Blue Black, Ruthenium Red, and Toluidine Blue. Samples were collected 24 h after the beginning of germination under salt stress conditions of 0, 4, and 8 dS m⁻¹. Scale bar: 100 μm. Magnification: 20X

Due to the absence of starch in the embryo section, we stained it with Aniline Blue Black, Ruthenium Red, and Toluidine Blue to determine the distribution of metabolites within the embryo, specifically at the junction where the initial part of the mesocotyl shoot meets the scutellum (Fig. 11). In this study, we also demonstrated changes in the cellular and intercellular concentrations of sugars, tannins, pectins, and proteins, focusing on metabolite variations between the coleoptile section of the mesocotyl at the onset of shoot development and the region where the coleoptile merges with the scutellum (Fig. 11). In salt stress, it is seen that tannins are more abundant in shoot and mesocotly cells due to the presence of more pink and purple colours (Fig. 11). However, in the absence of salt stress, the shoot and mesocotyl regions exhibited higher protein content. Furthermore, it was observed that density and mobility of protein was significantly greater in the shoot section. While the shoot was protein-rich at the onset of germination, it was also found that sugars, tannins, and pectins were present in the mesocotyl cells and the intercellular spaces (Fig. 11). As shown in Fig. 11, the cells in the shoot section are smaller than those in the squamous section. Additionally, the shoot cells are organized in a systematic arrangement.

### Degradation of proteins stored in the aleurone layer during germination stages

**Fig. 12:**
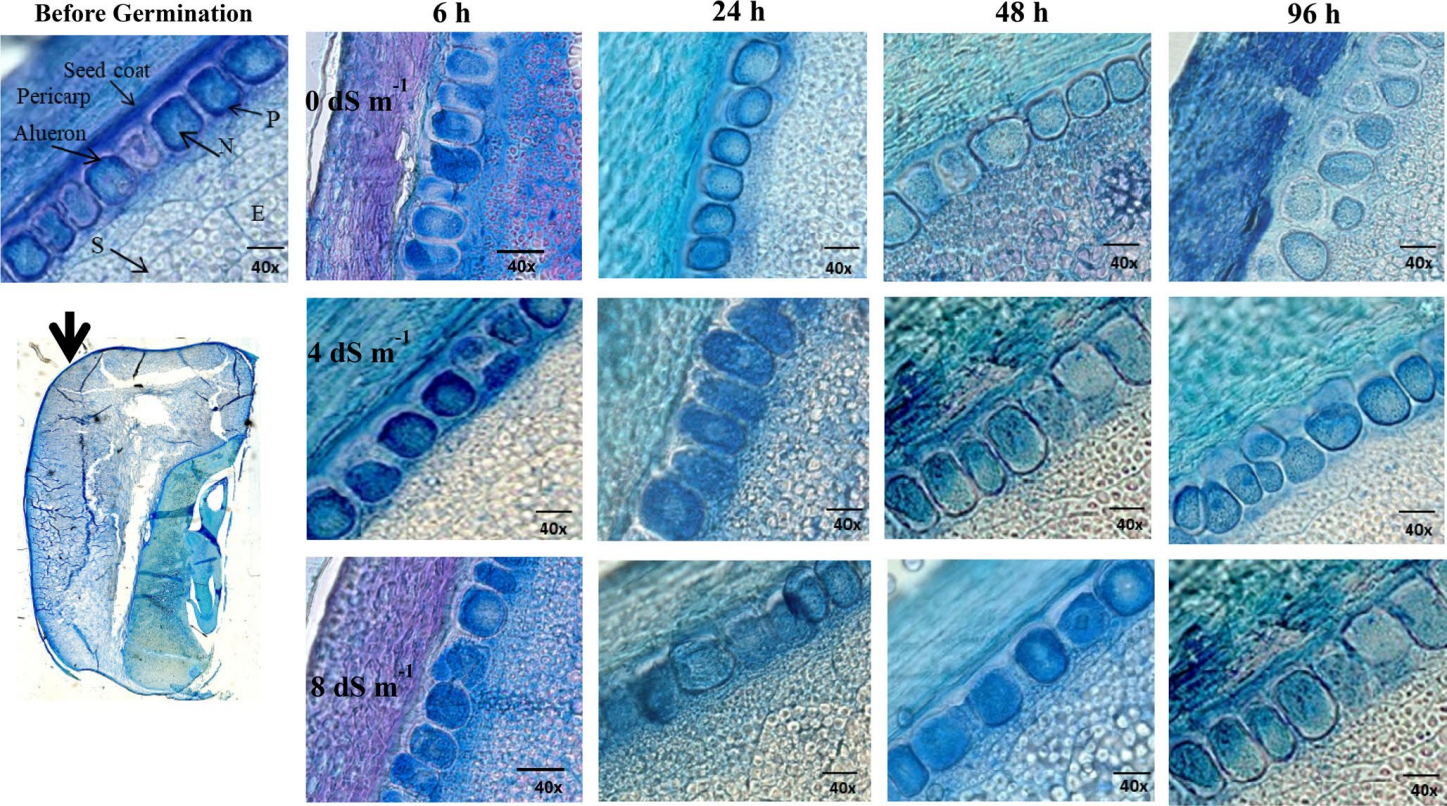
The light microscope illustrates the changes in the aleurone layer, seed coat, and endosperm of corn seeds during germination under salinity stress conditions of 0, 4, and 8 dS m⁻¹. (S): Starch granules, (N): Nucleus, (P): Protein. The sectionings were made precisely in the middle of the seed, and the photos were taken from the area indicated by the arrow. The images show seeds germinating under three different salt concentrations, progressing from left to right and from 6 h to 96 h

We determined that the aleurone is the outermost layer of the endosperm, consisting of specialized cells that are rich in proteins and enzymes (Fig. 12). In our study, as the germination process advanced, the proteins stored in the aleurone layer slowly underwent degradation. Interestingly, another finding in this study was that after fulfilling their role of producing enzymes and mobilizing nutrients, the aleurone cells did not undergo programmed cell death (Fig. 12). Of course, germination is a continuous process. It was possible that the death of these aleurone cells could occured at later stages of germination. The aleurone layer, which was responsible for producing hydrolytic enzymes that degrade stored starch, proteins, and lipids in the endosperm, was not negatively affected under salinity stress due to osmotic stress, ion toxicity, and oxidative stress (Fig. 12). In the absence of salt stress conditions, the proteins in the aleurone cells and just below the cell rapidly decrease as germination progresses. In contrast, with increasing concentrations of salt stress, the reduction of proteins in the aleurone cells occurs at a slower rate (Fig. 12). It was clearly observed that the shrinkage or degranulation of auleron cells begins at 96 h under non-salt stress condition (Fig. 12).

## Discussion

### Development of the radicle and changes of the intercellular space of pericarp cell (SEM)

As the seed absorbed water and begins to germinate, the pericarp gradually becomed thinner and more porous under non-stress condition. Interestingly, the irregular cracks that would typically result from salt stress on our seeds were not fully visible. Perhaps this can cause the testa to maintain its hardness, potentially delaying or inhibiting germination. Numerous structural changes in the testa indicated that salinity can cause mechanical stress on the seed coat, thereby hindering germination. The presence of these crystals, which appeared as white spots on the seed due to high salt concentration, might have also disrupted the normal functioning of the seed coat, thereby further inhibiting germination. Under salinity stress, the presence of inadequate pores or malformed micropyles in the seed coat hindered the diffusion of water and oxygen (Zhu et al. 2021). The onset and duration of water uptake in the seeds of certain plants can vary. In our study, particularly under non-stress conditions, we observed that the seeds absorbed water within the first six hours, resulting in changes to the cells of the intercellular space between the pericarp cells. Of course, one of the primary reasons for this phenomenon could be believed to be the open micropyle of the seed and the absence of salt stress. Cells may appear shrunken, collapsed, or disrupted, indicating (Renard et al. 2023) that salinity is affecting the internal structure of the seed coat. But in our study, The pericap, or outer layer, of the maize seed is nearly smooth before germination. In the absence of salt, the intercellular space between the pericarp cells open rapidly; however, this process occured much more slowly as salt stress increases. In addition, no significant deformation or cell death was observed in the pericarp or outer surface cells of corn seeds germinated under salt stress conditions of 4 and 8 dS m⁻¹. This may be because, although it is dent corn with a high starch content, the corn seed used is more vitreous and has a harder structure. The radicle root cap is a crucial structure that safeguards the growth tip of the radicle (the embryonic root) during seed germination (Berhin et al. 2019). Under salinity stress, the root cap encounters significant challenges that affect the overall success of seed germination and early plant development (Atta et al. 2023). In our study, salinity stress diminished the size of the root cap, thereby impairing its protective function. When pushing through the soil, the smaller root cap is less effective at protecting the growing tip from mechanical damage (Iijima et al. 2004). When exposed to elevated levels of salt, root cap cells were exhibit alittle disorganization or irregularity according to the results of SEM. This disorganization weakens the structural integrity of the cap, making it can be more susceptible to damage and less effective at guiding the radicle. To maintain its protective function as the radicle grows, the root cap naturally sheds old cells while new ones are produced (Goh et al. 2022). Although salinity can accelerate the premature shedding of the cells of the root cap, a process known as ‘sloughing’, in our study, the cells in the root cap were not completely shed by the 96th hour of exposure to 4 dS m⁻¹ salt stress. However, this does not imply that they will not be shed during subsequent periods of germination under salt stress. Since sampling was not conducted between the 48th and 96th hours in the study, it is possible that the root cap under non-stressed conditions could have been similar to the root cap of the seed subjected to 4 dS m^− 1^ salt stress at the 96th hour.

### The differences of meristematic cells and root development compared between salt stress and non-salt stress

Salinity impairs growth rates by disrupting the regulation of essential genes involved in the cell cycle (Bhat et al. 2024). The quiescent center was a small group of cells in the root meristem that divided infrequently but played a crucial role in maintaining the activity of the surrounding stem cells (Fig. 5). We think that as the seedling begins to grow and differentiate, new cellulose could be synthesized to strengthen the newly formed cells. This process should contributes to the formation of new primary cell walls in the rapidly dividing and expanding cells of the embryo. Of course, insufficient water uptake could delay or inhibit radicle emergence. In our study, we also observed that the root cells of the seeds germinated under salt stress did not develop even after 96 h. Therefore, we can not exactly compare the effect of salinity stress. Perhaps, high salinity could lead to lignification (thickening) of xylem cell walls, which restricts the flow of water and nutrients (Le Gall et al. 2015; Böhm et al. 2010) or the Casparian strip, a barrier located within the endodermis, may become more rigid in response to increased salinity, thereby limiting the movement of ions (Chen et al. 2011).

### The biochemical structure of cells in the embryo (The middle of schutullem) during germination

The acidic polysaccharides, such as pectins and mucopolysaccharides, undergo significant changes that play a crucial role in embryo development, seedling emergence, and cell wall remodeling during seed germination (Haas et al. 2021). Pectins are a group of complex acidic polysaccharides that serve as major components of plant primary cell walls and middle lamellae (Voragen et al. 2009). They explained also the pectins play a crucial role in maintaining the structure, adhesion, and porosity of the cell wall. In addation, the energy in dormant seeds is mainly stored as polysaccharide starch granules in the endosperm, which are mobilized during germination by hydrolytic enzymes like α- and β-amylases released by the aleurone layer and scutellum (Zeeman et al. 2007). We can think that the reason why during germination of the corn seed, pectins are degraded by pectinases, including polygalacturonases and pectin methylesterases, which depolymerize the pectin matrix (Lionette et al. 2017). In our study, excessive breakdown of pectin led to insufficient degradation, which could impede germination under saline conditions (Fig. 6). Conversely, under conditions without salt stress, this depolymerization is believed to soften the cell wall, enabling the radicle (embryonic root) to break through the seed coat (testa) and initiate seedling development in the absence of stress. Additionally, our study revealed that both pectins and mucopolysaccharides play a crucial role in modifying the mechanical properties of cell walls, thereby facilitating cell growth due to the structural changes in the cell walls of embryonic cells (Fig. 6). Hence, under non-stressed conditions, the softening of the seed coat through enzymatic activity on these polysaccharides allows the radicle to penetrate, marking the visible onset of germination, because intense metachromatic staining highlighted cells with dense cytoplasm and small vacuoles, indicating elevated metabolic activity at this stage. Therefore, remodeling of cellulose was essential for loosening the cell wall, which facilitates cell expansion and the emergence of the radicle (the embryonic root) (Barnes and Anderson 2018). This is partly accomplished by modifying the cellulose network. We believe that enzymes, such as cellulases, are activated during germination and partially break down cellulose microfibrils, making cell walls more flexible and capable of expansion. Therefore, the cell walls must be loosened to allow the seed embryo to grow and for the radicle to emerge. But the result of study showed that in contrast to pectin, cellulose was not fully degraded during germination. Our study also demonstrated that cell walls can stretch without compromising their structural integrity, as the breakdown is partial and localized. This ability may be crucial for allowing the radicle to penetrate the testa.

One additional finding in the study was that tannins degraded or leached during the initial stages of germination. During germination, enzymes such as polyphenol oxidases and peroxidases break down these complexes, increasing the permeability of seed tissues and facilitating the mobilization of nutrients (Waskow et al. 2021). Because tannins can form complexes with proteins or polysaccharides, stabilizing the seed’s tissues and protecting them during dormancy (Shirley 1998). They can protect emerging seedlings from oxidative stress by neutralizing free radicals, which are often produced in response to environmental stresses during germination (Tuladhar et al. 2021). As observed in our study, tannins are broken down at a slower rate under salinity stress. This may be because tannins still play a role as antioxidants (Tong et al. 2022). There are two important facts: the breakdown of cellulose releases sugars that can be utilized by the developing embryo, while the reduction of tannins enhances the bioavailability of proteins, starches, and other reserves stored in the seed.

### The changes of starch and protein in embrio and endosperm cells

In the storage organs of plants, starch serves as the primary form of carbohydrate and energy reservoir (Ma et al. 2020). The amylolytic degradation of starch stored in the endosperm of seeds during different stages of germination is a crucial biochemical and biophysical process that provides reducing sugars as energy sources to support the initial stages of seed germination in angiosperm plants (Chen et al. 2019). In our study clearlly showed that starch stored in the seed are locazied in the endosperm, while not any starch granule in embrio (Figs. 7, 8 and 9). During germination, starch is broken down to generate energy and metabolites (Tang et al. 2017). Several studies have revealed that sucrose, which is one of the most important substrate sources for the formation of storage reserves, acts as a primary nutrient and energy source for plant development (Wang et al. 2016; Ma et al. 2020). As we considered, the activated enzymes hydrolyze starch, breaking it down into smaller sugars. These sugars were transported to the developing embryo to serve as energy sources for cellular respiration and as building blocks for cell growth.

The our study showed that proteins were stored in specialized organelles called protein bodies or vacuoles, particularly in the embrio (Scutullem). Upon imbibition (water uptake), proteolytic enzymes, such as proteases, are activated to break down storage proteins into their constituent amino acids. This process is often regulated by hormones, particularly gibberellins (GA), which stimulate the synthesis of proteases (Gao and Chu 2020). During this phase, storage proteins, including globulins, albumins, prolamins, and glutelins (depending on the seed type), are hydrolyzed (Gil et al. 2024). In cereal seeds such as wheat and barley, glutelins and prolamins are major storage proteins (Shewry and Halford 2002). The hydrolysis of storage proteins releases amino acids into the embryo, which are then used for the synthesis of new proteins essential for the growing seedling. In our study, we think that due to the high rate of protein turnover—where old or unnecessary proteins were degraded during germination—new proteins were synthesized to meet the needs of the developing seedling. Therefore, proteins were consistently present in cells under both non-stress and salt stress conditions. In addation, particularly under unfavorable environmental conditions (e.g., salinity, drought, cold), seeds may synthesize stress-related proteins to protect the embryo from damage during germination (Marthandan et al. 2020). Therefore, during the early stages of seed germination, storage proteins could be rapidly degraded, on the contrary the total soluble protein content could be increased as new enzymes and functional proteins were synthesized at all stage of germination both contidions. In another study explained that salt stress conditions could increase an oxidative burst characterized by excessive accumulation of H₂O₂, resulting in cell death due to significant damage to biomolecules such as lipids and proteins (Sofo et al. 2015). However, in our study, no deaths or complete destruction were observed in the scutellum and endosperm cells of germinating maize under extreme salt stress.

It has been observed that the degradation of starch occurs at a significantly slower rate in seeds germinated under salt stress conditions. This suggests that, for cellular respiration to generate ATP—the energy currency of the cell—the energy provided by the seed through starch remains at very low levels. It is important to consider that during the initial imbibition phase of seed germination, when the seed absorbs water, metabolic activity and enzyme activation are delayed or hindered due to osmotic stress and low turgor pressure. Consequently, the enzymes required to break down starch cannot function effectively, thereby halting the germination process under saline conditions. The poor seed quality of this type of maize can be attributed to a small endosperm, a small embryo, high sugar content, and low starch levels in the endosperm. These factors render the maize susceptible to fungal and soil-borne pathogens (Styer and Cantliffe 1984).

Although we could not exactly identify the oil bodies in our study, it is thought that the white oil bodies can seen especially in embryo cells are oil droplets (Fig. 6). Zhu et al. (2023) explained that fatty acids transition during germination stages II and IV after water uptake, demonstrating that free fatty acids are released from triacylglycerol and subsequently degraded to acetyl-CoA, accompanied by the release of energy and carbon during the germination of Zostera marina seeds. They noted that the levels of organic acids (succinic, fumaric, citric, and isocitric) and ATP increased significantly during stages III and IV, indicating that the TCA cycle produced numerous intermediates (organic acids). Pirahanchi and Sharma (2024) explained that upon imbibition (water uptake) and the initiation of germination, lipase enzymes become activated.

### Degradation of the proteins stored in aleurone layer during germination stages

Rajjou et al. 2012 showed that the endosperm serves as the primary source of energy and nutrient substrates essential for the growth and development of the embryo. Programmed cell death (PCD) occurs in the cells of the aleurone layer to enhance the provisioning process. More energy and substrates may be necessary not only for germination but also for resisting salinity stress. Wang et al. 2024 explained that during seed germination, starch, is broken by Amylases (e.g., α-amylase and β-amylase) triggered by the hormone gibberellin (GA), which signals the aleurone layer to secrete amylase into the starchy tissues in the endosperm or cotyledons, down into simple sugars such as glucose, maltose, and sucrose.

Salinity stress significantly affected the breakdown of starch into simple sugars, which can critical for providing energy during germination in our study. There may be many reasons such as inhibition of imbibition, delayed enzyme activation, reduced α-amylase activity, hormonal imbalance, decreased glucose and maltose levels during germination under salinity. The fluidity and transfer of sugars from the endosperm to the embryo developing could be often impaired under salt stress. This reduces the availability of energy in the embryo, slowing down the development of key structures like the radicle (embryonic root) and plumule (shoot). Once sugars such as glucose, maltose, and sucrose are produced in the endosperm, they are transported to the growing embryo through plasmodesmata (cytoplasmic channels) or via apoplastic (cell wall) and symplastic (cytoplasm-connected) pathways (Aoki et 2012). Or The sugars, particularly sucrose, are actively transported from the endosperm to the embryo by sugar transporters situated in the cell membranes of both endosperm and embryo cells (Lafon-Placette and Köhler 2014). Aubert et al. 2018; the aleurone cells begin to produce a variety of enzymes that assist in mobilizing storage reserves in the starchy endosperm once they are activated by gibberellins. The aleurone layer’s response to gibberellins (GAs) and abscisic acid (ABA) is crucial for seed germination (Gómez-Cadenas et al. 2001). Salt stress increases ABA levels, which inhibit GA-mediated α-amylase secretion. This, in turn, impacts the mobilization of starch reserves. The reason the auleron layer was not completely destroyed in our study may be that the plant shoot had not yet begun photosynthesis.

## Conclusion

Salinity stress induces various changed in the aleurone layer and its proteins, primarily decreasing the production of enzymes essential for nutrient mobilization. The aleurone layer played a crucial role in supporting the germination of maize seeds by breaking down nutrients stored in the endosperm and making them available to the developing embryo. Protein dynamics during seed germination involved the degradation of storage proteins to supply amino acids, the activation of pre-existing proteins, and the synthesis of new proteins necessary for metabolism, growth, and stress responses. The transfer of sugars from the endosperm to the embryo during seed germination is a complex and tightly regulated process that is essential for seedling growth. The osmotic and ionic stress caused by salinity disrupts water uptake, inhibits the activity of essential starch-degrading enzymes, and alters sugar transport and utilization. Sugars were mobilized from stored starch, oligosaccharides, and lipids to supply the energy and carbon necessary for growth during seed embryo germination. As the germination of the seed embryo, cellulose was partially degraded to facilitate the loosening and expansion of the cell wall, which is essential for the emergence of the seedling. The tannins were reduced or broken down to eliminate their inhibitory effect on germination, while simultaneously playing a protective role in the early stages. Salt stress induced various levels of metabolic disruption during seed germination, impacting essential processes such as nutrient mobilization, energy production, and protein synthesis, which ultimately hindered seedling growth and development. The hormonal, enzymatic, and biochemical changes in the structures within the seed under conditions of salt stress compared to non-stress conditions exhibited a complex and distinct nature. In this study, we have analyzed the biochemical transformations occurring in various seed structures, including the seed coat, aleurone layer, embryo, and endosperm, and how these structures respond to salt stress. Although some of the results in our study align with findings in the existing literature, others differ due to the genetic variations among the maize genotypes used as seed material. Notably, the grain structure may exhibit a more glassy texture. Consequently, it is essential to investigate the structural and chemical differences in maize grains, as well as the biochemical changes occurring in the cellular structures of different seed parts during the germination stages of flour corn, sweet corn, horse tooth corn, and popcorn.

## Data Availability

No datasets were generated or analysed during the current study.
